# *PLOS Biology* at 20: Ain’t no mountain high enough

**DOI:** 10.1371/journal.pbio.3002011

**Published:** 2023-01-27

**Authors:** Nonia Pariente, on behalf of the PLOS Biology staff editors

**Affiliations:** Public Library of Science, San Francisco, California, United States of America and Cambridge, United Kingdom

## Abstract

As PLOS Biology enters its third decade, we reflect on our mission, what has changed, what remains to be done and our wishes for the future.

## Teaser

PLOS began publishing influential open access science in 2003. As *PLOS Biology* enters its third decade, we reflect on our mission, what has changed, what remains to be done and our wishes for the future.

This article is part of the *PLOS Biology* 20th Anniversary Collection.

2003 was a remarkable year. The world marveled at the completion of the Human Genome Project, Twitter and Facebook did not yet exist and, as the first Chinese manned space mission took flight, so too did *PLOS Biology* “to demonstrate that high-quality journals can flourish without charging for access” [1]. This year, we celebrate 20 years of publishing high-quality science that is available to everyone in the world.

Although our work is not finished, the progress of the past two decades has been nothing short of remarkable. When PLOS launched in 2000, Harold Varmus, Michael Eisen and Patrick Brown called for articles to be made freely available 6 months after publication —an idea so radical that it failed to gain traction, prompting PLOS to become a publisher to demonstrate what was possible. Now, major funders across the world, such as the signatories of Plan S and the US Office of Science and Technology, request immediate open access (OA) publication for the research that they fund. In 2003, when *PLOS Biology* appeared on the scene, only a handful of life science journals were OA. The picture is dramatically different now: the Directory of Open Access Journals lists 18,881 journals and >8.5 million articles (up from 1.5 million 10 years ago [2]), and in 2020 more articles were published OA than behind paywalls for the first time, according to data from the Dimensions database. We are proud of our role in starting the ripple effect that has led to this unstoppable wave.

In the years following the launch of its flagship, *PLOS Biology*, PLOS launched more journals, including its clinical flagship, *PLOS Medicine*, and *PLOS ONE*, the world’s first multidisciplinary peer-reviewed journal to focus on rigorous research and ethics rather than conceptual advance. The whole PLOS portfolio adheres to principles of open science (OS) that go beyond OA, including, for example, the requirement that all underlying data be made freely available upon publication. While we see OA as a good beginning, full access to the results of scientific research, as well as to the code, software and materials generated, and to the methodological details that enable reproduction should be the direction of travel.

The growth and increased acceptance of preprint servers in the life sciences in the past ten years has also shaken up research communication. Cold Spring Harbor Laboratory launched bioRχiv in 2013 and medRχiv in 2019, and there are now over 50 platforms covering biology and/or medicine. We were early supporters, and now most journals accept preprint submissions and even integrate preprint servers into their workflows, as we do by allowing bidirectional posting to bioRχiv. Major funders and institutions recognize preprints as evidence of productivity in grant, hiring and tenure applications, which has boosted the practice. In the future, some form of (pre)publication, followed by peer review (journal-associated or journal-independent, by the community and/or by individuals recruited for that purpose) and curation seems a likely scenario for scientific publishing; eLife’s recent changes to their publishing model are a move in this direction.

Nice as it is to see how far the needle has moved in transforming research communication, our work is not done. For example, OA has moved the access barrier to publication rather than to reading an article, which is a problem that we have not yet solved. PLOS is actively trying to address this issue through innovative publishing models, including *PLOS Biology*’s Community Action Publishing.

Improvements to the peer review process and making it sustainable in the face of increasing research output remains an important pain point. We are actively engaging with journal-independent peer review through platforms such as Review Commons and Peer Community In, as well as championing portable peer review for articles that have had an initial assessment elsewhere and continue the process with us, rather than starting from scratch. We have had positive experiences so far, but the implementation of a large cross-publisher agreement to share reviewer reports and identities (with their permission) or journal-independent review at a system-wide scale would avoid futile peer review cycles that waste reviewer and researcher time.

As a community, we need better ways of crediting different research outputs and of crediting individual contributions to articles that have ever-larger authorship teams. We also need systemic change in how we fund science and hire/promote researchers, moving away from perverse incentives based on journal-level metrics, which reinforce practices that decrease the overall reliability of the scientific output. We should be assessing the specific contributions made by an applicant’s work and, importantly, the robustness and reliability of said work. However, systemic change towards more reproducible research will not occur until researchers are incentivized to invest the time and resources required for OS, which are currently undervalued or not considered at all. This should change.

Admittedly, the robustness, transparency or reliability of research is not easy to measure and easy-to-use proxies are often required to allocate scarce resources/positions when there are large numbers of applications. Article-level metrics, which PLOS introduced in 2009, are an improvement over the journal impact factor for assessing a specific scientific contribution. However, measuring the openness and reproducibility of research outputs is currently not straightforward. To help address this problem, PLOS, in collaboration with DataSeer, has recently developed a suite of Open Science Indicators to measure OS practices. We expect that developments in this space, such as the community consensus on core open science practices to monitor in biomedicine that we publish in this issue [3], will soon allow funders and institutions to reward these practices. As the COVID-19 pandemic has shown, OS enables faster scientific progress, which should be, after all, the ultimate goal of research.

In addition to developing ways of signaling trustworthiness and reliability, we will need to curate the ever-increasing corpus of scientific literature (currently ca. 2 million articles per year in the life and health sciences, and growing), while facing challenges such as the growing presence of paper mills. This is why we believe that selectivity is a useful editorial criterion and one we plan to continue to uphold (although, as we’ve discussed previously, our take on selectivity differs from the norm, emphasizing the research question asked rather than the results [4]).

In 20 years’ time, when the *PLOS Biology* editors of the future take stock, we hope they see progress on these fronts and that we have been part of the solution.

As we look to the future, in addition to our more traditional scope, we also want to draw attention to areas of keen societal relevance, such as planetary health and global change biology, emerging infectious diseases, antimicrobial resistance, metabolism and metabolic disease, aging and age-related diseases, neural disease, and cancer, as well as rapidly developing areas such as biotechnology and structural biology. Meta-research will also remain a key focus for *PLOS Biology*—research on how research is designed, performed, communicated, used and assessed is more important than ever if we are to design data-driven strategies to address some of research’s pressing issues.

During this year of celebration, reflection and inspiration, we have commissioned Perspectives that look back at landmark *PLOS Biology* papers and reflect on their contribution to the field. The first two [5, 6] cover the first transcriptome of the form of the malaria parasite that infects red blood cells, which was published in our inaugural issue [7], and an influential method for inferring evolutionary time from molecular phylogenies from 2006 [8]. We will also publish Perspectives in which authors take stock of the past two decades in a given field and look forward to what is coming next. In October, our anniversary month, we will devote our magazine section to discussing some of the most pressing issues in scientific publishing.

As we begin our third decade, we would like to hear from you, our readers, authors, reviewers and editors. As we said in our first issue, “PLoS Biology is yours. Download it, copy it, incorporate it in your own database, post or reprint any article; use your imagination. We ask only that you give fair credit to the authors of any work that you use” [9]. This is your journal—it is with you and for you that we work to transform research communication.
